# Supplementary material for: Individual retrotransposon integrants are differentially controlled by KZFP/KAP1-dependent histone methylation, DNA methylation and TET-mediated hydroxymethylation in naïve embryonic stem cells
Source: Epigenetics Chromatin. 2018 Feb 26;11:7. doi: 10.1186/s13072-018-0177-1 (PMC6389204; doi:10.1186/s13072-018-0177-1)
Supplement: Supplementary file 11 — Additional file 11. Pattern analysis. [file 13072_2018_177_MOESM11_ESM.zip › Patterns analysis/DataTables/extensions/Scroller/examples/simple.html]

Scroller example - Basic initialisation


# Scroller example Basic initialisation

Scroller is a plug-in for DataTables which enhances DataTables' built-in scrolling features to allow
large amounts of data to be rendered on page very quickly. This is done by Scroller through the use of
a virtual rendering technique that will render only the part of the table that is actually required for
the current view.

Note that Scroller assumes that all rows are of the same height (in order to preform the required
calculations. You can use `td { white-space: nowrap; }` in your CSS to ensure that text in
rows does not wrap.

This example shows how Scroller for DataTables can be initialised by simply including the character
`S` in sDom (note that the `S` must come after the `t` in sDom).
Deferred rendering an and Ajax data source are also used in this example.

| ID | First name | Last name | ZIP / Post code | Country |
| --- | --- | --- | --- | --- |

- Javascript
- HTML
- CSS
- Ajax
- Server-side script

The Javascript shown below is used to initialise the table shown in this
example:

`$(document).ready(function() {
$('#example').DataTable( {
ajax: "data/2500.txt",
deferRender: true,
dom: "frtiS",
scrollY: 200,
scrollCollapse: true
} );
} );`

In addition to the above code, the following Javascript library files are loaded for use in this
example:

- ../../../media/js/jquery.js
- ../../../media/js/jquery.dataTables.js
- ../js/dataTables.scroller.js

The HTML shown below is the raw HTML table element, before it has been enhanced by
DataTables:

This example uses a little bit of additional CSS beyond what is loaded from the library
files (below), in order to correctly display the table. The additional CSS used is shown
below:

The following CSS library files are loaded for use in this example to provide the styling of the
table:

- ../../../media/css/jquery.dataTables.css
- ../css/dataTables.scroller.css

This table loads data by Ajax. The latest data that has been loaded is shown below. This data
will update automatically as any additional data is loaded.

The script used to perform the server-side processing for this table is shown below. Please note
that this is just an example script using PHP. Server-side processing scripts can be written in any
language, using the protocol described in the
DataTables documentation.

## Other examples

### Examples

- Basic initialisation
- State saving
- Client-side data source (50,000 rows)
- Server-side processing (5,000,000
  rows)
- API

Please refer to the DataTables documentation for full
information about its API properties and methods.  
Additionally, there are a wide range of extras and
plug-ins which extend the capabilities of
DataTables.

DataTables designed and created by SpryMedia Ltd © 2007-2014  
DataTables is licensed under the MIT license.
